# Visual Image Annotation for Bowel Obstruction: Repeatability and Agreement with Manual Annotation and Neural Networks

**DOI:** 10.1007/s10278-023-00825-w

**Published:** 2023-06-06

**Authors:** Paul M. Murphy

**Affiliations:** 1 University of California–San Diego, 9500 Gilman Dr, 92093 La Jolla, CA USA; 2 UCSD Radiology, 200 W Arbor Dr, 92103 San Diego, CA USA

**Keywords:** Bowel obstruction, Eye tracking, Segmentation, Quantification, Convolutional neural networks

## Abstract

Bowel obstruction is a common cause of acute abdominal pain. The development of algorithms for automated detection and characterization of bowel obstruction on CT has been limited by the effort required for manual annotation. Visual image annotation with an eye tracking device may mitigate that limitation. The purpose of this study is to assess the agreement between visual and manual annotations for bowel segmentation and diameter measurement, and to assess agreement with convolutional neural networks (CNNs) trained using that data. Sixty CT scans of 50 patients with bowel obstruction from March to June 2022 were retrospectively included and partitioned into training and test data sets. An eye tracking device was used to record 3-dimensional coordinates within the scans, while a radiologist cast their gaze at the centerline of the bowel, and adjusted the size of a superimposed ROI to approximate the diameter of the bowel. For each scan, 59.4 ± 15.1 segments, 847.9 ± 228.1 gaze locations, and 5.8 ± 1.2 m of bowel were recorded. 2d and 3d CNNs were trained using this data to predict bowel segmentation and diameter maps from the CT scans. For comparisons between two repetitions of visual annotation, CNN predictions, and manual annotations, Dice scores for bowel segmentation ranged from 0.69 ± 0.17 to 0.81 ± 0.04 and intraclass correlations [95% CI] for diameter measurement ranged from 0.672 [0.490–0.782] to 0.940 [0.933–0.947]. Thus, visual image annotation is a promising technique for training CNNs to perform bowel segmentation and diameter measurement in CT scans of patients with bowel obstruction.

**Supplementary Information:**

The online version contains supplementary material available at 10.1007/s10278-023-00825-w.

## Introduction

Bowel obstruction is a common cause of acute abdominal pain [1], found in approximately 15% of emergency department presentations for that indication. Abnormalities such as adhesions, hernias, or tumors block transit of contents through the gastrointestinal tract, resulting in dilation of the upstream bowel [2]. Imaging such as radiographs and CT help determine the severity and etiology of the obstruction to guide management decisions [3]. On these modalities, obstruction is suggested if the diameter of the bowel exceeds approximately 3 cm for the small bowel or 6 cm for the large bowel [4, 5]. It is differentiated from adynamic ileus by the absence of distal bowel gas and by the presence of a transition point in the caliber of the bowel, at which the cause of the obstruction can often be identified.

Automated detection and characterization of bowel obstruction may help guide diagnosis and management. Development of such methods requires annotation of imaging studies for training and validation. Manual annotation can be time-consuming, due to the large number of voxels within cross-sectional imaging studies that must be labeled for applications such as segmentation. Fortunately, other approaches based on the use of eye tracking devices have been developed [6–9]. These approaches leverage the cognitive skill of radiologists to identify anatomic or pathologic structures within an imaging volume. Eye tracking devices can record in real time the location on a monitor at which a radiologist casts their gaze. If these gaze locations can be used in place of manual annotations, it may accelerate the process of image annotation.

This study investigates visual annotation of two aspects of the bowel relevant to obstruction: measurement of its diameter and segmentation between foregut, midgut, and hindgut. The diameter of the bowel determines whether a segment qualifies as dilated. The threshold for dilation depends on whether the dilated segment is part of the midgut or of the hindgut. Repeatability between two sessions of visual annotation can be quantified with the Dice coefficient for segmentation [10] and with intraclass correlation and limits of agreement for diameter measurements [11–13]. These same quantities can be calculated to assess the agreement between visual and manual annotations. These metrics provide information about the suitability of visual annotations for subsequent applications. The first aim of this study is to determine the repeatability of visual annotation and to assess its agreement with manual annotation for bowel segmentation and diameter measurement.

Convolutional neural networks (CNNs) promise to expand the range of bowel diseases for which automated detection and characterization on medical imaging is possible [14, 15]. Some imaging studies of the gastrointestinal tract, as well as the classic image processing algorithms used for their interpretation, require specific preparation of the bowel prior to the study. For instance, in CT colonography, the colon must be adequately insufflated with gas, after a sufficient bowel preparation, to increase the visual and algorithmic conspicuity of polyps [16–20]. Insufficient preparation reduces the performance of CT colonography algorithms due to their reliance on specifically encoded features of the input data.

In other contexts, such as CT scans performed for acute abdominal pain, there may be no opportunity for such preparation, resulting in a more heterogeneous appearance of the bowel and its contents [2]. Similarly, patients imaged for acute abdominal pain often have a wide range of comorbidities, resulting in a heterogeneous appearance of the remainder of the peritoneal cavity as well. These clinical and technical features pose challenges to the automated detection and characterization of bowel obstruction, one common cause of such presentations.

Fortunately, neural networks do not rely upon specifically encoded features of the input data, but rather, they can learn from annotated training data regardless of its heterogeneity [21–25]. In addition, even the quality of the annotations need not be perfect in every instance, since neural networks can learn to perform well on average over the entire training data set [26]. However, a critical requirement for neural network development is the availability of large sets of annotated data for training and validation.

Visual annotation with an eye tracker may serve to generate such data sets. The approach described in this study can be used to annotate bowel segmentation and diameter maps in a large number of subjects. CNNs can then be trained to predict these features of importance in bowel obstruction. Agreement can be quantified with the Dice coefficient for bowel segmentation [10] and with intraclass correlation and limits of agreement for diameter measurements [11–13]. If their predictions are sufficient for downstream applications, CNNs may contribute to the diagnosis of this common cause of acute abdominal pain. The second aim of this study is to assess the agreement of CNN predictions with visual and manual annotations of bowel segmentation and diameter measurements in CT scans of subjects with bowel obstruction.

## Materials and Methods

### Study Design

IRB approval was granted for this HIPAA-compliant study with a waiver for informed consent due to its minimal risk. There was no overlap in the subject population with previously published results and no conflict of interest.

### Population

Patients from a single adult tertiary care center were retrospectively included if they had a CT scan of the abdomen and pelvis with a clinical report containing in its impression the phrase “bowel obstruction.” This criterion captured reports where that phrase was prefaced by the words “small,” “large,” or “no” to ensure that the data set included examples of dilated and non-dilated segments across the gastrointestinal tract. The impression rather than indication for the CT was used to ensure inclusion of an adequate number of scans with obstruction. Such scans may have been ordered for an indication of abdominal pain, but obstruction is found in only a fraction of scans for that indication. CT scans were included regardless of whether their clinical context was emergent, inpatient, or outpatient.

Consecutive CT scans between March and June 2022 were included until the population reached 50 subjects, a sample size feasible for subsequent annotation. Some CTs contained multiple phases, and some subjects had multiple CT scans, yielding 60 scans in total. Only thick slice axial reconstructions were included. Scans were deidentified prior to annotation.

Relevant clinical and technical features were characterized on each scan as present or absent or as another dichotomy if appropriate: sex; jejunal, ileal, or colonic dilation; bowel resection; ostomy; hernia; abundance or paucity of mesenteric fat; peritoneal or mesenteric malignancy; ascites; pneumoperitoneum; and intravenous or enteric contrast. Subjects were partitioned to minimize the sum squared difference in the prevalence of these features between training and test data sets. Subjects with more than one scan were excluded from the test data set.

### Visual Annotation

Each scan was visually annotated using a Tobii 4c eye tracker and a custom-developed module of 3D Slicer [27]. The eye tracker generated data at 60 Hz corresponding to the location on the monitor at which a radiologist cast their gaze. The module transformed those gaze locations, along with the slice position, averaged over 0.75 s intervals, into 3-dimensional coordinates within the imaging volume. A region of interest (ROI) was depicted at the gaze location within the imaging volume, with a diameter that was adjusted with a rotary encoding knob.

For each scan, a series of segments was recorded until the entire gastrointestinal tract had been covered. For each segment, a radiologist cast their gaze at the centerline of the bowel. They adjusted the diameter of the ROI to approximate the diameter of the bowel. They adjusted the slice location with a mouse scroll wheel to navigate along the length of the bowel, for as long as its course was apparent. Each segment was assigned a label indicating to which of 13 parts of the gastrointestinal tract it belonged: esophagus; stomach; duodenum; jejunum; ileum; cecum; appendix; ascending, transverse, descending, and sigmoid colon; rectum; and anus.

**Fig. 1:**
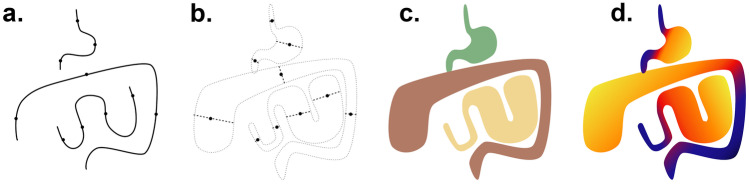
Diagram of visual annotation. A series of gaze points representing the centerline of the bowel were recorded with an eye tracker (**a**). An approximate diameter of the bowel was also recorded for each gaze point (**b**). For segmentation, the region of the imaging volume closest to each segment was assigned the label of that segment (**c**). For the diameter map, each voxel of the imaging volume was assigned the diameter of the closest segment (**d**)

The series of segments was transformed into volumetric segmentations and diameter maps through a geometric model (Fig. 1). For each voxel, the closest segment was identified. If the distance was within one-half of the diameter of the segment, the voxel value was set to the corresponding label or zero otherwise. The diameter map was defined similarly, except that voxel values were set to the diameter of the closest segment. Segmentations were shown superimposed on the CT scan in 3D Slicer and were updated after each segment was recorded for real-time feedback (see Supplemental Information video).

Calibration of the eye tracker was performed using the vendor’s software prior to recording. Fine adjustments to the calibration could be made based on offsets of the mouse position. The gaze location within the imaging volume was centered in the viewport of the 3D Slicer window to reduce the range of the screen over which calibration was needed.

A radiologist (PM) with 10 years of experience performed all annotations. Visual annotations were repeated two times for each scan with minimal intervening time between repetitions. Both repetitions of visual annotation were included in the training and test data sets. Visual and subsequent manual annotations were performed during different sessions separated by at least 1 week.

### Manual Annotation

Each scan was manually annotated. Segments of the bowel that were *en face* to the axial plane were chosen for each of the 13 parts of the gastrointestinal tract listed above if they were present in each scan. A pair of calipers was placed on the short and long axes of those segments in 3D Slicer. Four pairs of calipers were placed on different segments of the jejunum and ileum in different locations throughout the quadrants of the abdomen.

Since the time required for manual segmentation of the entire imaging volume would have been prohibitive, a single slice of the imaging volume was randomly chosen. All bowel present on that slice was segmented by tracing its margin in 3D Slicer and was assigned one of 3 labels: foregut (esophagus through duodenum), midgut (jejunum and ileum), or hindgut (cecum through anus).

These divisions were intended to correspond to thresholds for obstruction rather than embryologic definitions [28]. The embryologic foregut includes the duodenum only to the level of the ampulla, but the parts of the duodenum had not been labeled to allow such a division. Since at baseline, the duodenal bulb can be larger than the threshold for small bowel obstruction, the duodenum was included with the foregut for purposes of this study. Likewise, the embryologic hindgut includes only the distal colon, but the proximal colon was included with the hindgut as well, due to similar thresholds for obstruction.

### Data Normalization and Augmentation

Data were normalized prior to training. CT scans were scaled to a range of [−1,1] using a window of 400 HU and a level of 40 HU. The 13-part segmentation of the entire gastrointestinal tract was reduced to a 3-part segmentation corresponding to foregut, midgut, and hindgut. The diameter maps initially expressed diameter in units of millimeters but were divided by the voxel dimensions of the scan to express diameter as a number of voxels instead. This conversion was performed since the field of view of CT scans varies in size, as do patients themselves, which may confound diameter prediction in units of millimeters. On the other hand, measurements in units of voxels are consistent across scans and patients and can be converted to millimeters subsequently, by multiplication with the known voxel dimensions. The volumes were resized from a matrix size of 512 × 512 voxels to 256 × 256 voxels for the 2d model or to 128 × 128 × 64 voxels for the 3d model. Since the CT scans varied in their number of slices, they were zero padded in that direction prior to resizing for the 3d model so that no interpolation was needed.

Data augmentation was performed using random translations between ± 30 mm along the x and y axes; random rotations between ± 30° around the x, y, and z axes; and random magnifications of ± 30%. The values of the diameter map were scaled by the same magnification factor so that diameters expressed as a number of voxels would be consistent after augmentation. Augmentation was performed in a way that preserved geometry despite the anisotropy of the voxels of the thick slice CT scans. The number of augmentations was chosen such that the training data set filled nearly the entirety of system memory (32 GB).

### Neural Networks

**Fig. 2:**
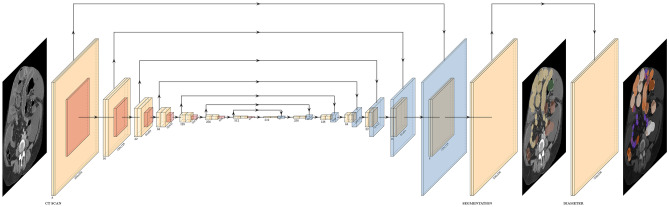
Diagram of CNN topology. CNNs were constructed from multiple blocks in a U-net topology. Convolutional, maxpool, and upsampling layers in each block are depicted in yellow, red, and blue, respectively. Batch normalization and dropout layers were also included in each block, and a softmax layer was included in the segmentation output, but these are omitted from this figure for simplicity. Direct and skip connections between blocks are depicted as arrows. Dimensions were either halved or doubled between each block and are listed below each convolutional layer. For the 2d CNN, the initial dimensions were 256 × 256 voxels by 8 channels, and the U-net had 14 blocks. For the 3d CNN, the initial dimensions were 128 × 128 × 64 voxels by 16 channels, and the U-net had 10 blocks, to maintain a similar number of parameters. The input of the CNN was the CT scan, and the outputs were a segmentation and a diameter map

Two CNNs were constructed in Keras/Tensorflow version 2.7.0 [29] based on the U-net topology [23, 30]: a 2d model for which the input was a slice of the scan and a 3d model for which the input was the entire scan (Fig. 2, adapted from [31]). Each level of the model consisted of two convolutional layers, two activation layers, a maxpool layer in the descending limb or an upsampling layer in the ascending limb, a batch normalization layer, a dropout layer, and feedforward connections between levels of equal shape in the descending and ascending limbs. The number of levels was selected such that the midpoint of the network was 2 × 2 in shape. The number of filters in each level was selected such that the 2d and 3d models had a similar number of parameters (approximately 8 M and 9 M), and both fit in GPU memory (12 GB) using 16-bit floating point precision. Two model outputs were generated from the final level of the network: a segmentation output via a softmax layer and a diameter output via another activation layer.

The loss function was the sum of three terms: categorical cross-entropy of the 3-part segmentation, binary cross entropy of the dichotomized segmentation, and a mean squared error of the diameter, weighted to approximately the same order of magnitude as the first two terms.

$$L_{CE}=-{\textstyle\sum_{k=0}^3}\;t_k\log\left(p_k\right)$$

$$L_{BCE}=-t_0\log(p_0)-(1-t_0)\log(1-p_0)$$

$$L_{SE}={(d_t-d_p)}^2$$

$$t_k=true\;probability\;for\;label\;k$$

$$p_k=predicted\;probability\;for\;label\;k$$

$$d_t=true\;diameter$$

$$d_p=predicted\;diameter$$

The dichotomized segmentation between the background and any of the foregut, midgut, or hindgut was intended to favor differentiation from the background even when differentiation among parts of the gut was ambiguous. A sample weight was applied to increase the contribution to the loss function of all voxels that were non-zero in the training data. Ten percent of the training data set was reserved for validation. The test data set was not used for either training or validation of the models. Models were trained on a single NVIDIA Titan XP GPU for 100 epochs with the Adam optimizer, a learning rate of 1e-3, and randomly initialized parameters. The model with the best validation loss was saved. The predictions were resized to the initial CT scan dimensions for subsequent analysis.

### Statistical Analysis

Summary statistics including total, mean, and standard deviation were calculated. The prevalence of each relevant clinical and technical feature was calculated. The length of each segment was calculated as the sum of the distance between sequential gaze points. For segmentations, Dice scores were calculated as the ratio of the size of the intersection to the sum of the sizes of the components [10]. For diameter measurements, intraclass correlation was calculated using the “irr” package and “icc” function for two-way agreement in R version 4.1.2 [32]. Ninety-five percent limits of agreement were also calculated [13]. Both repetitions of visual annotation were included in the comparisons with manual annotations and CNN predictions.

## Results

### Summary Statistics

Summary statistics of 60 CT scans of 50 subjects included in the study are given in Tables 1 and 2.

**Table 1:** Clinical and technical features of subjects and scans in the entire data set and in the set of subjects reserved for testing using a denominator equal to the number of scans (*n* = 60 or 10)

	**All**	**Test**
**Sample size**		
* Subjects*	50	10
* CT scans*	60	10
**Demographics**		
* Sex (F)*	0.50	0.40
* Age (years)*	F:59.2, M:60.4	F:61.9, M: 56.8
* Age range (years)*	19–90 +	33–76
**Clinical features**		
* Jejunal dilation*	0.62	0.70
* Ileal dilation*	0.34	0.30
* Colonic dilation*	0.10	0.10
**Surgical features**		
* Hernia*	0.16	0.20
* Bowel resection*	0.38	0.40
* Ostomy*	0.32	0.30
**Peritoneal contents**		
* Abundant mesenteric fat*	0.50	0.50
* Paucity of mesenteric fat*	0.38	0.40
* Peritoneal malignancy*	0.18	0.20
* Ascites*	0.22	0.20
* Pneumoperitoneum*	0.06	0.10
**Technical features**		
* Intravenous contrast*	0.88	0.80
* Enteric contrast*	0.16	0.10
**Manual annotations**		
• **All scans, one repetition**		
* All*	1102	178
* Foregut*	192	32
* Midgut*	483	81
* Hindgut*	427	65

**Table 2:** Summary statistics of technical features and annotations. Total, mean, or standard deviations are reported using a denominator equal to the number of scans (*n* = 60) except where indicated

**CT scan dimensions**	
* Axial matrix size*	512
* Axial voxel size (mm)*	0.75 ± 0.09
* Axial FOV (cm)*	38.6 ± 4.9
* Number of slices*	127.9 ± 16.9
* Slice thickness (mm)*	3.72 ± 0.26
* Longitudinal FOV (cm)*	47.3 ± 5.1

**Visual annotations**	
• **Per segment**	
* Gaze points*	14.3 ± 7.0
* Recording time (sec)*	10.7 ± 5.3
* Length (mm)*	97.5 ± 61.6
• **Per scan**	
* Segments*	59.4 ± 15.1
* Gaze points*	847.9 ± 228.1
* Recording time (min)*	10.6 ± 2.9
* Length (meters)*	5.8 ± 1.2
• **All scans, two repetitions**	
* Segments*	7131
* Gaze points*	101,743
* Recording time (hours)*	21.2
* Length (kilometers)*	0.7

The prevalence of each clinical and technical feature was similar between the entire data set and the set of subjects partitioned into the test data set, which ensured that examples of each feature would be available for both training and testing. Both dilated and non-dilated segments of the jejunum, ileum, and colon were included, which ensured that the full range of relevant diameters would be present for each part of the gastrointestinal tract. The prevalence of features such as paucity of mesenteric fat, peritoneal malignancy, and ascites reflects the complexity of the subjects included in this study. The distribution of manual annotations across the foregut, midgut, and hindgut was approximately 3:8:7 in the test data set, similar to the entire data set.

Overall, 59.4 ± 15.1 segments, 847.9 ± 228.1 gaze locations, and 5.8 ± 1.2 m of bowel were recorded per scan. The large number of short segments reflects the fact that much of the bowel could be followed for only a short distance before its course became inapparent.

Since each gaze location represented 0.75 s of the recording time of the eye tracker, the total amount of time used to record gaze locations was 10.6 ± 2.9 min per scan. However, that quantity underestimates the total time required to annotate each scan, since it does not include the time required for the radiologist to deduce the course of the bowel, calibrate the eye tracker, inspect the results, redo inaccurate recordings, or manage interruptions. That total time was not feasible to record in the scope of this study.

The length of several meters of bowel per scan is of the same order of magnitude as the known length of the gastrointestinal tract of humans [33]. Recorded lengths may be lower due to the exclusion of most of the esophagus from the field of view of the CT scans or due to prior bowel resections in the population included in this study.

In total, 1102 pairs of calipers were placed across the gastrointestinal tract over all 60 scans for manual annotation of bowel diameter. Annotations were distributed in an approximate 3:8:7 ratio through the foregut, midgut, and hindgut, which was intended to reflect a combination of the length and subjective clinical importance of each part of the gastrointestinal tract in the context of bowel obstruction.

### Examples of Annotations and Predictions

**Fig. 3:**
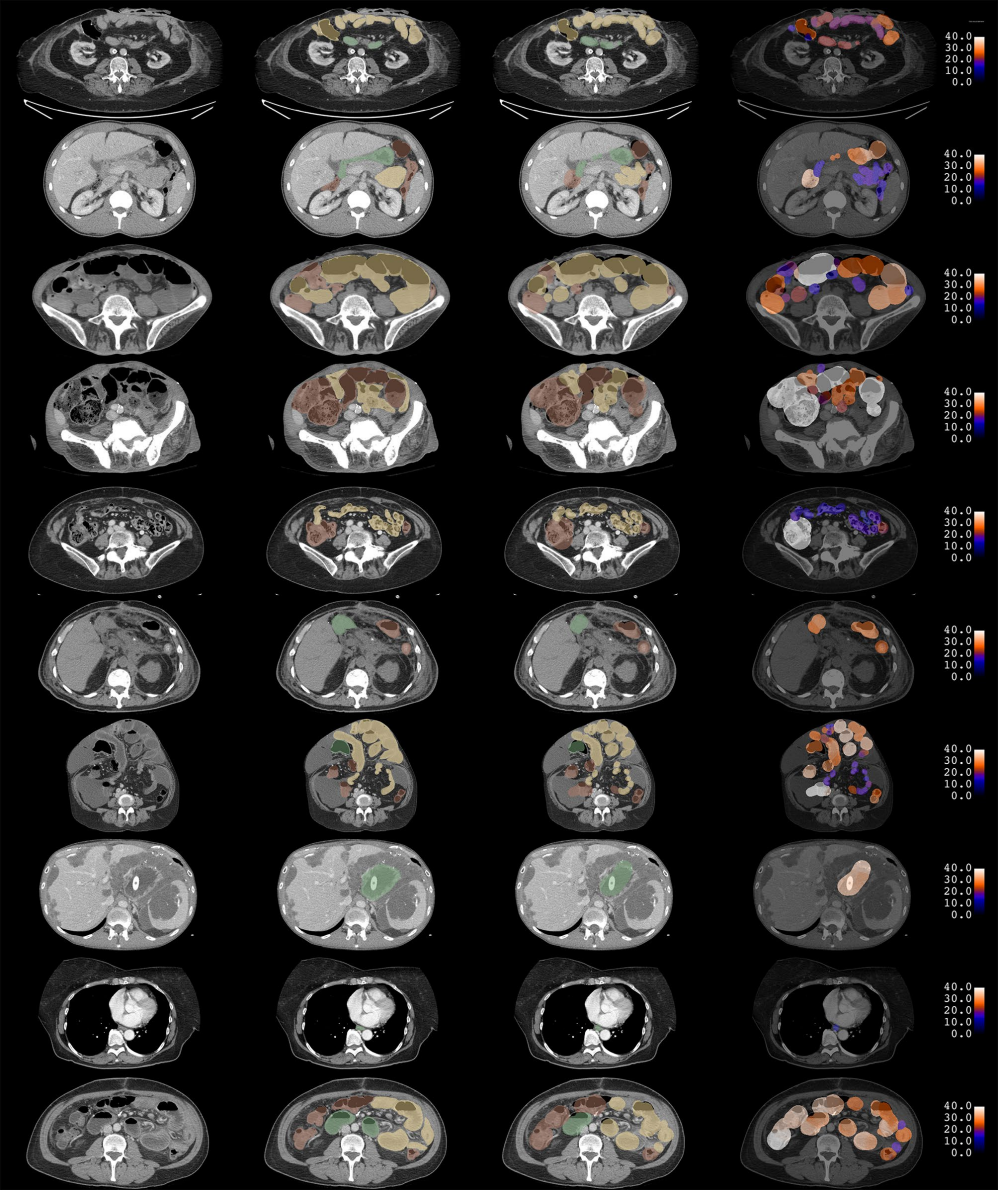
Examples of manual and visual annotation. Each row corresponds to a single slice of a CT scan for 10 different subjects. The CT scan is shown in the first column. Manual segmentations are shown in the second column. Segmentations from visual annotation are shown in the third column. In both segmentations, red, yellow, and green correspond to foregut, midgut, and hindgut, respectively. Diameter maps from visual annotation are shown in the fourth column. Diameters are expressed in units of millimeters. Manual diameter measurements are not shown

**Fig. 4:**
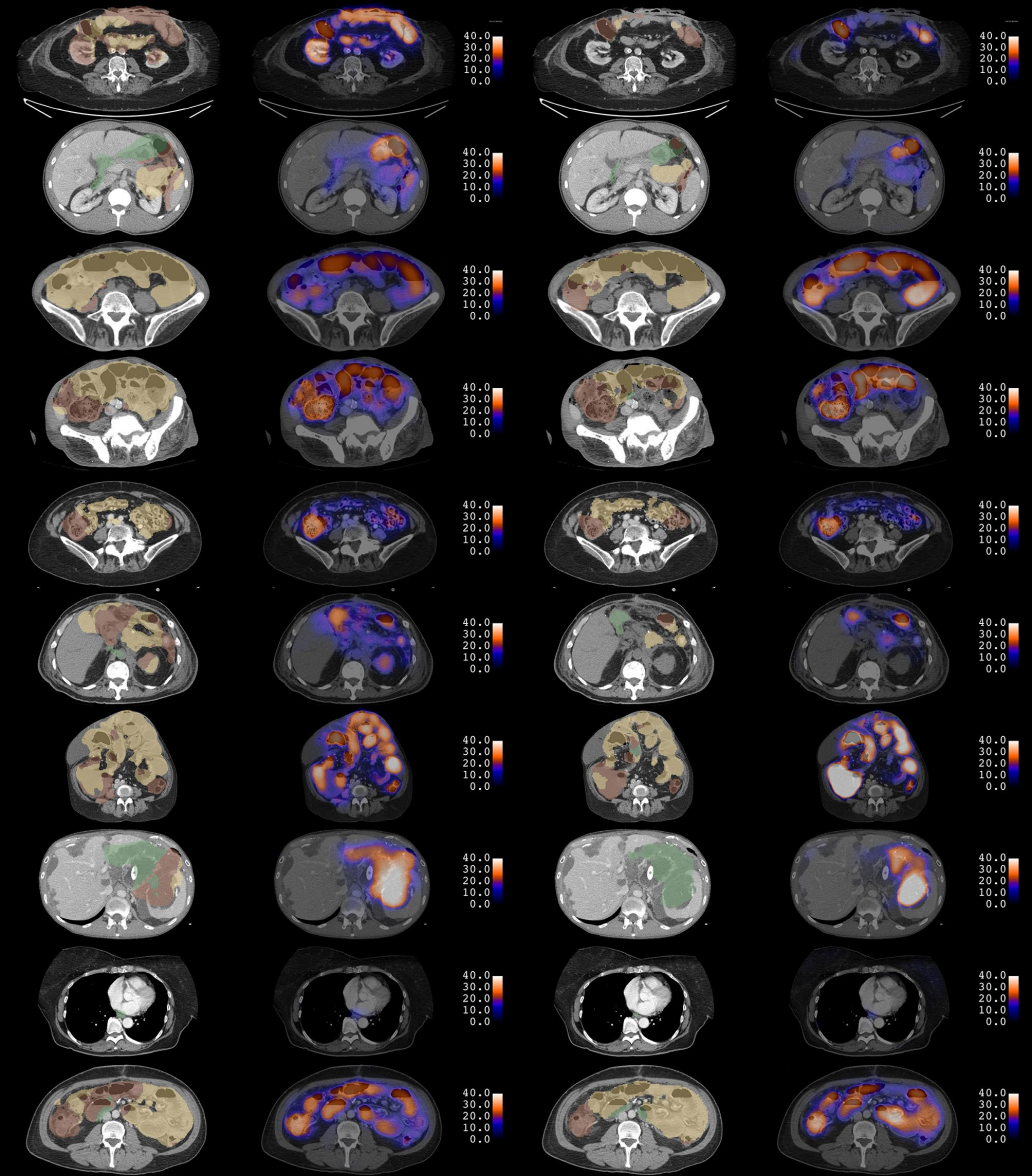
Examples of CNN predictions in 10 test subjects. Each row corresponds to the same single slice of a CT scan as in Fig. 3. 2d CNN predictions for bowel segmentation and diameter maps are shown in the first and second columns. 3d CNN predictions are shown in the third and fourth columns. In both segmentations, red, yellow, and green correspond to foregut, midgut, and hindgut, respectively. Diameters are expressed in units of millimeters. Some incorrect predictions of features are noted

Examples of manual and visual annotations and CNN predictions for ten subjects in the test data set are shown in Figs. 3 and 4.

### Agreement of Visual Segmentations

**Fig. 5:**
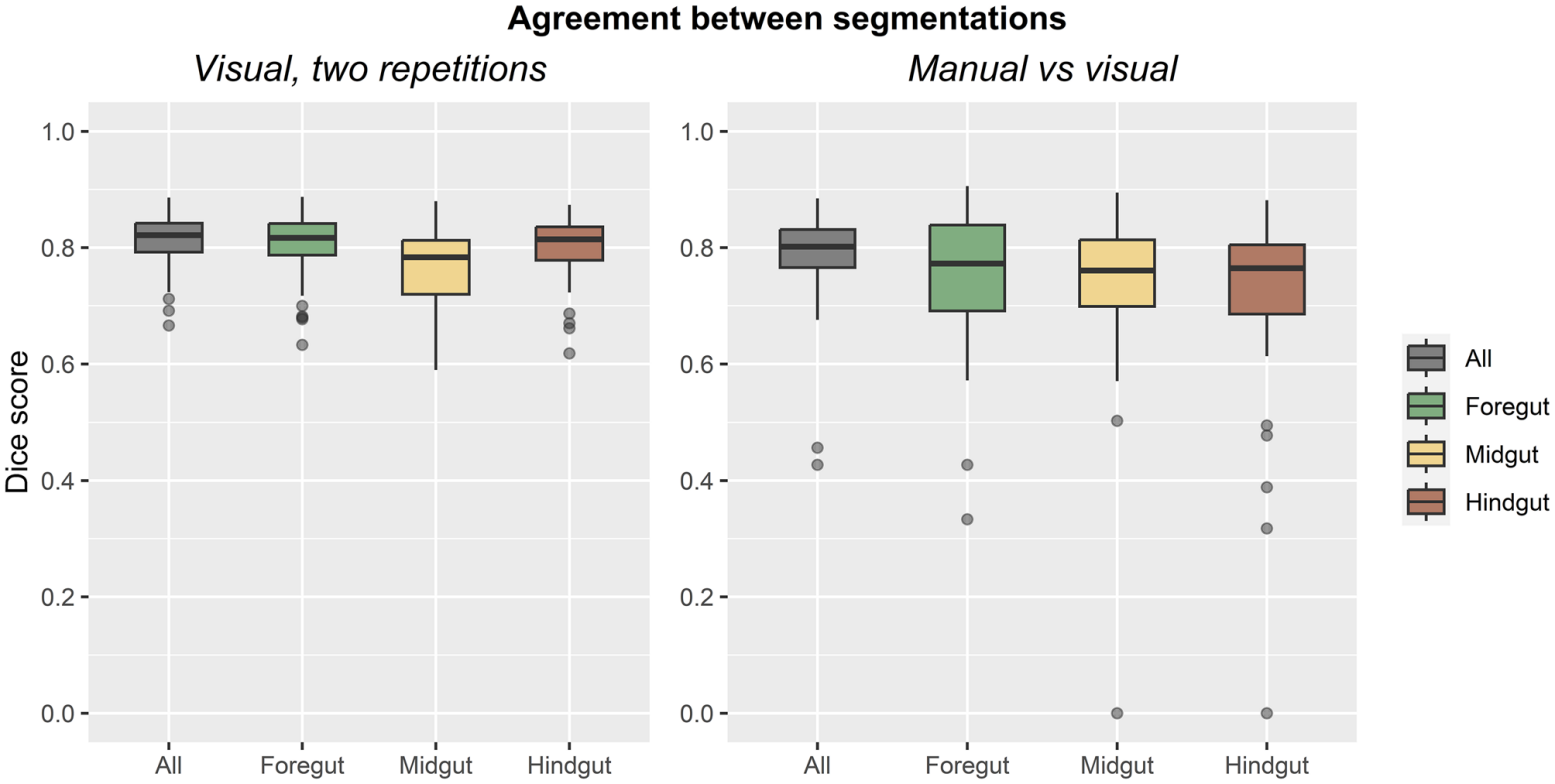
Agreement of segmentations. Boxplots are shown for Dice scores between two repetitions of visual annotation (left) and for manual versus visual annotations (right)

Dice scores for all segmentations are reported in Table 3 and shown in Fig. 5. Between two repetitions of visual annotation, Dice scores were 0.81 ± 0.04 for the entire gastrointestinal tract. Dice scores were slightly lower for the foregut, midgut, and hindgut separately, ranging from 0.77 to 0.81, which may reflect variations in the boundaries at which each part was annotated.

**Table 3:** Agreement of Dice scores for segmentation between two repetitions of visual annotations and between manual and visual annotations. Means, standard deviations, and sample sizes are reported

***Dice score***	**Visual, two repetitions**	**Manual versus visual**
*All*	0.81 ± 0.04 (*n* = 60)	0.79 ± 0.08 (*n* = 60)
*Foregut*	0.81 ± 0.06 (*n* = 60)	0.74 ± 0.13 (*n* = 29)
*Midgut*	0.77 ± 0.06 (*n* = 60)	0.74 ± 0.14 (*n* = 44)
*Hindgut*	0.80 ± 0.05 (*n* = 60)	0.72 ± 0.16 (*n* = 51)

Between manual and visual annotations, Dice scores were 0.79 ± 0.08 for the entire gastrointestinal tract. Similarly, Dice scores were lower for the foregut, midgut, and hindgut separately, ranging from 0.72 to 0.74. Dice scores could not be calculated if a part of the gastrointestinal tract was absent from the single slice chosen for manual annotation. For instance, if a slice through the pelvis was chosen, the foregut may be absent from both manual and visual annotations for that slice. Therefore, such slices were excluded from the calculations for that part, and sample sizes were lower for the foregut, midgut, and hindgut separately than they were for the entire gastrointestinal tract.

Overall, these Dice scores represent good repeatability of visual annotation and good agreement with manual annotations.

### Agreement of Visual Diameter Measurements

**Fig. 6:**
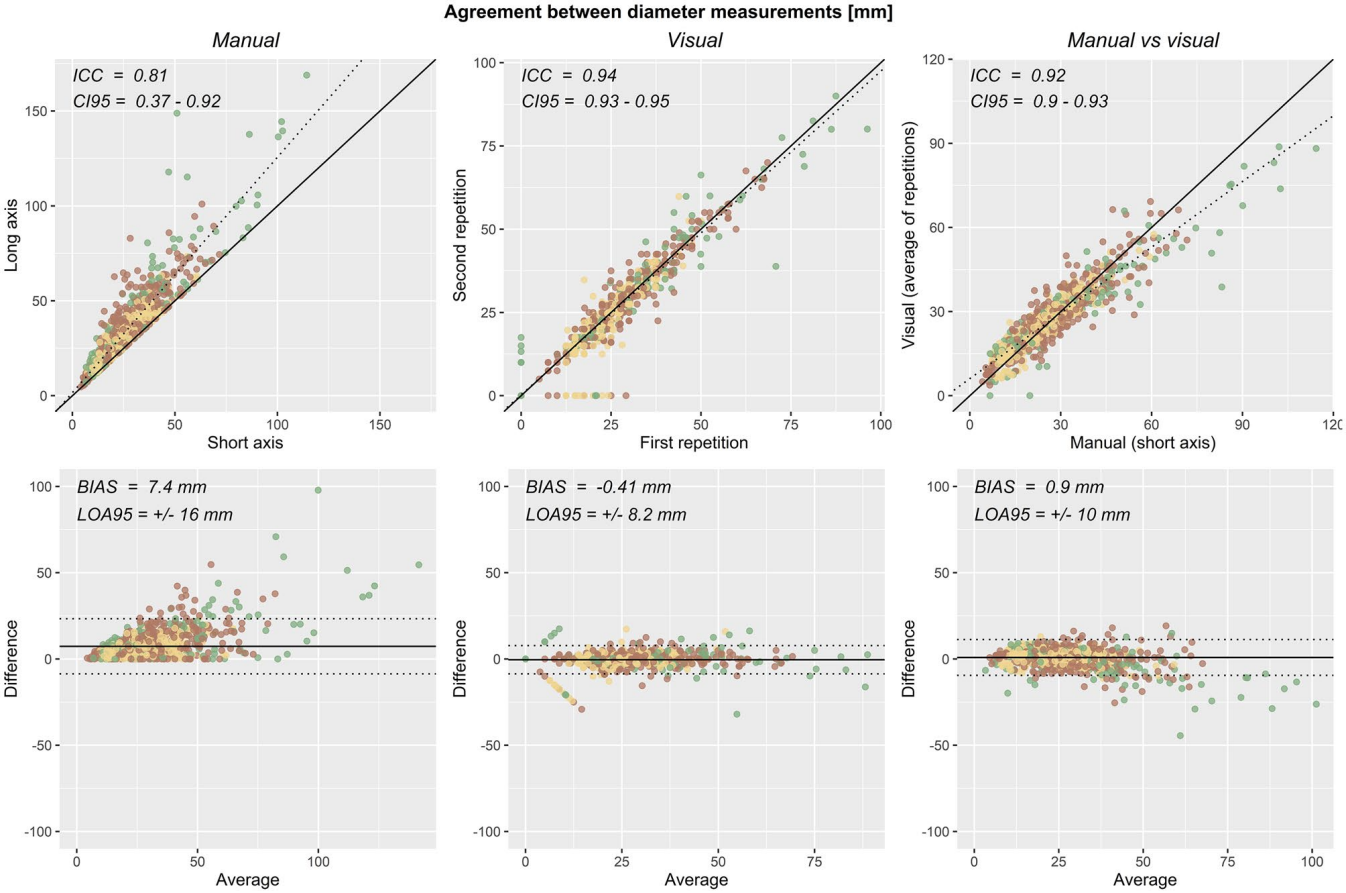
Agreement of diameter measurements. Scatterplots (top) and Bland–Altman plots (bottom) of diameter measurements are shown for manual annotation of short- versus long-axis measurements (left), for two repetitions of visual annotation (middle), and for manual versus visual annotations (right)

Intraclass correlation and 95% limits of agreement for diameter measurements are reported in Table 4 and shown in Fig. 6. Between two repetitions of visual annotation, intraclass correlation and 95% limits of agreement were 0.940 [95% CI = 0.933–0.947] and −0.41 ± 8.2 mm across the entire gastrointestinal tract. Between manual and visual annotation, intraclass correlation and 95% limits of agreement were 0.917 [95% CI = 0.905–0.927] and 0.9 ± 10 mm across the entire gastrointestinal tract.

**Table 4:** Agreement of diameter measurements made with manual and visual annotations. Intraclass correlation and its 95% confidence interval, as well as bias and 95% limits of agreement, are reported

**Comparison**	**ICC [95% CI]**	**Bias ± 95% LOA**	**Sample size**
*Manual, short axis vs long axis*	0.807 [0.366–0.915]	7.4 ± 16 mm	1102
*Visual, two repetitions*	0.940 [0.933–0.947]	−0.41 ± 8.2 mm	1102
*Manual vs visual*	0.917 [0.905–0.927]	0.9 ± 10 mm	1102

As a comparison, the intraclass correlation and 95% limits of agreement were 0.807 [0.366–0.915] and 7.4 ± 16 mm for manual measurements of the short-axis as compared with the long-axis diameters. Thus, manual and visual diameter measurements agreed better than short-axis and long-axis diameters measured manually, even though measurements were taken at segments that were *en face* to the axial plane.

Overall, there was excellent repeatability of visual diameter measurements and excellent agreement with manual annotations.

### Agreement of CNN Segmentations

**Fig. 7:**
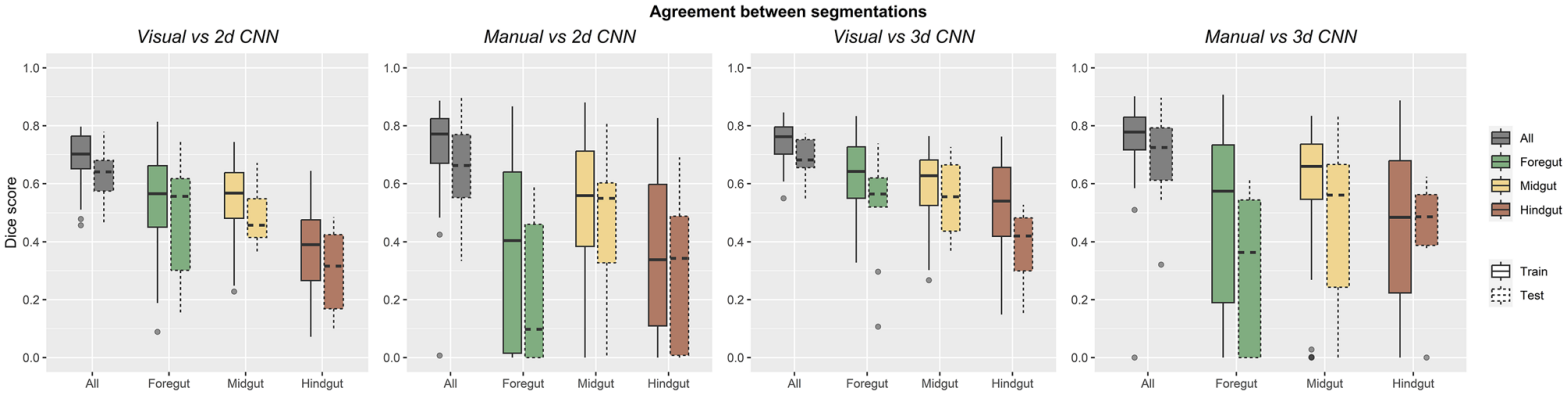
Agreement of segmentations. Boxplots are shown for Dice scores between 2 and 3d CNN predictions and visual and manual annotations for the training and test data sets

Dice scores between segmentations are reported in Table 5 and shown in Fig. 7. For 3d CNN segmentations of the entire gastrointestinal tract over the 10 CT scans of the test data set, Dice scores were 0.69 ± 0.07 compared to visual annotations and 0.69 ± 0.17 compared to manual annotations. As expected, Dice scores were higher over the 50 CT scans of the training data set than over the test data set, 0.75 for both visual and manual annotations. Although these Dice scores reflected only moderate agreement, they would not be expected to exceed the Dice scores between two repetitions of visual annotation or between visual and manual annotations.

**Table 5:** Agreement of 2d and 3d CNN predictions with visual and manual annotations over the training and test data sets. Mean, standard deviation, and sample size of Dice scores for segmentation are reported

**2d CNN**	**Visual**		**Manual**	
***Dice score***	**Train**	**Test**	**Train**	**Test**
* All*	0.69 ± 0.09 (*n* = 50)	0.63 ± 0.10 (*n* = 10)	0.73 ± 0.15 (*n* = 50)	0.65 ± 0.20 (*n* = 10)
* Foregut*	0.54 ± 0.17 (*n* = 50)	0.49 ± 0.21 (*n* = 10)	0.39 ± 0.32 (*n* = 24)	0.22 ± 0.26 (*n* = 8)
* Midgut*	0.55 ± 0.13 (*n* = 50)	0.48 ± 0.09 (*n* = 10)	0.50 ± 0.28 (*n* = 44)	0.44 ± 0.28 (*n* = 9)
* Hindgut*	0.38 ± 0.14 (*n* = 50)	0.30 ± 0.14 (*n* = 10)	0.35 ± 0.27 (*n* = 48)	0.30 ± 0.26 (*n* = 9)
**3d CNN**	**Visual**		**Manual**	
***Dice score***	**Train**	**Test**	**Train**	**Test**
* All*	0.75 ± 0.06 (*n* = 50)	0.69 ± 0.07 (*n* = 10)	0.75 ± 0.14 (*n* = 50)	0.69 ± 0.17 (*n* = 10)
* Foregut*	0.64 ± 0.12 (*n* = 50)	0.52 ± 0.19 (*n* = 10)	0.48 ± 0.33 (*n* = 27)	0.31 ± 0.27 (*n* = 8)
* Midgut*	0.59 ± 0.13 (*n* = 50)	0.55 ± 0.13 (*n* = 10)	0.59 ± 0.24 (*n* = 37)	0.47 ± 0.30 (*n* = 8)
* Hindgut*	0.51 ± 0.18 (*n* = 50)	0.38 ± 0.14 (*n* = 10)	0.45 ± 0.30 (*n* = 46)	0.44 ± 0.20 (*n* = 8)

Dice scores were lower for segmentations of the foregut, midgut, and hindgut, over both the training and test data sets, reflecting considerable variation in segmentation of each part of the gastrointestinal tract.

Dice scores were higher for the 3d CNN than for the 2d CNN, for both the training and test data sets, and across all parts of the gastrointestinal tract. This suggests that despite the lower in-plane resolution of the 3d CNN, which was 128 × 128 × 64, compared to the 2d CNN, which was 256 × 256, contextual information provided by adjacent slices was helpful for segmentation.

### Agreement of CNN Diameter Measurements

**Fig. 8:**
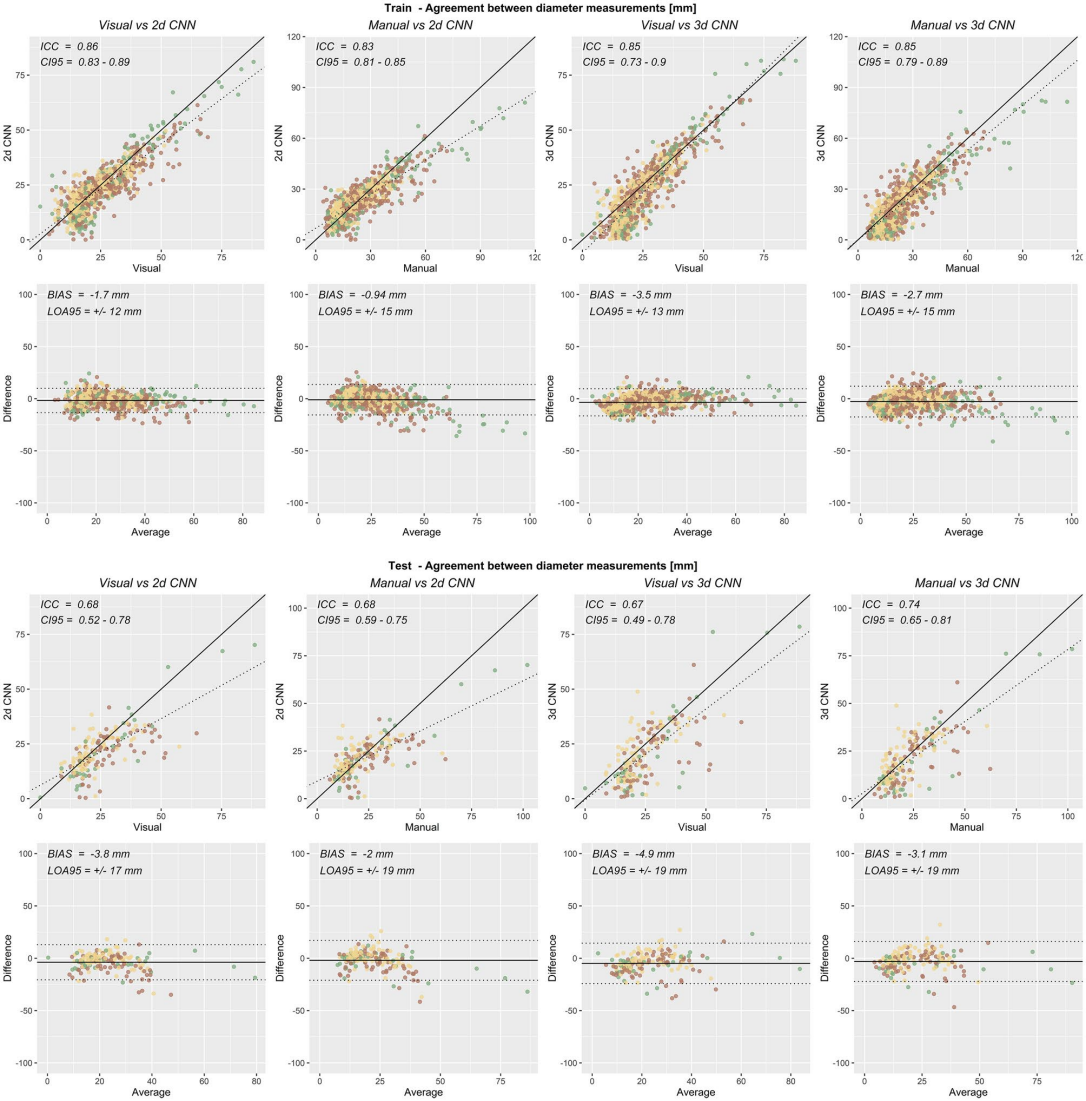
Agreement of diameter measurements. Scatterplots (top) and Bland–Altman plots (bottom) of diameter measurements are shown for 2d and 3d CNN predictions and visual and manual annotations over the training and test data sets

Intraclass correlations and 95% limits of agreements for diameter measurements are reported in Table 6 and shown in Fig. 8. For 3d CNN predictions over the 178 segments of the bowel in the test data set, intraclass correlation and 95% limits of agreement were 0.672 [95% CI = 0.490–0.782] and −4.9 ± 19 mm compared to visual annotations and were 0.739 [95% CI = 0.646–0.808] and −3.1 ± 19 mm compared to manual annotations. The slightly higher correlation with manual annotation than visual annotation suggests that the CNN may produce better measurements than existed in the data it was trained upon, which is possible since visual annotations did not agree perfectly with manual annotations.

**Table 6:** Agreement of CNN predictions with visual and manual annotations over the training and test data sets. Intraclass correlation and its 95% confidence interval, as well as bias and 95% limits of agreement, are reported for diameter measurements

**CNN**	**Annotation**	**Subjects**	**ICC [95% CI]**	**Bias ± 95% LOA**	**Sample size**
*2d*	*Visual*	Train	0.859 [0.825–0.885]	−1.7 ± 12 mm	924
		Test	0.675 [0.520–0.775]	−3.8 ± 17 mm	178
	*Manual*	Train	0.830 [0.808–0.850]	−0.94 ± 15 mm	924
		Test	0.677 [0.586–0.751]	−2 ± 19 mm	178
*3d*	*Visual*	Train	0.847 [0.729–0.904]	−3.5 ± 13 mm	924
		Test	0.672 [0.490–0.782]	−4.9 ± 19 mm	178
	*Manual*	Train	0.850 [0.794–0.887]	−2.7 ± 15 mm	924
		Test	0.739 [0.646–0.808]	−3.1 ± 19 mm	178

Intraclass correlations with manual annotations over the test data set were slightly higher for the 3d CNN than for the 2d CNN but were similar for visual annotations and for the training data set. This suggests that the contextual information available from adjacent slices was more important than in-plane resolution for diameter measurement.

The 95% limits of agreement were in the range of 1–2 cm for all comparisons, which compares unfavorably to the relevant diameter ranges in the context of bowel obstruction. Overall, the agreement of CNN predictions with visual and manual diameter measurements was only moderate.

## Discussion

These results show that visual image annotation with an eye tracker can be used for bowel segmentation and diameter measurement in CT scans of subjects with bowel obstruction, with good to excellent agreement over two repetitions and with manual annotations. These results also show that convolutional neural networks can be trained to perform bowel segmentation and diameter measurements in CT scans of subjects with bowel obstruction, with moderate agreement to visual and manual annotations. Whether such agreement is acceptable ultimately depends on downstream applications.

### Previous Studies

Eye tracking has been used in radiology for analysis of the patterns of eye motion during diagnostic evaluation of cancers on mammograms [34, 35], nodules on chest CTs [36, 37], and polyps on CT colonography [38–40]. More recently, eye tracking has also been used for the annotation of abnormalities on chest radiographs and brain tumors on MRI for subsequent deep learning [6–8]. This study expands the usage of eye tracking for annotation of the gastrointestinal tract for similar analyses and applications. Neural networks have also been used to accelerate image annotation with fewer manual interactions [41, 42]. Similar approaches may allow better use of eye tracking data than the geometric model of this study.

Diameter maps have been described for the characterization of biliary diseases [43]. In that study, classic image processing techniques were used to convert magnetic resonance cholangiopancreatography (MRCP) images into diameter maps. Metrics derived from these diameter maps were proposed as disease markers. The results of this study expand the usage of diameter maps to the gastrointestinal tract, and similar metrics may be derived for bowel obstruction.

Although Dice scores for automated segmentation exceed 0.9 for some abdominal organs [44, 45], the gastrointestinal tract presents additional challenges because of its variability. The results of this study are similar to prior studies, where Dice scores for segmentation of the gastrointestinal tract ranged from 0.55 to 0.88 [45–51]. However, direct comparison is limited due to differences in the modality [50, 51], the technique of the CT acquisition [47, 49], the part of the gastrointestinal tract that was segmented [45], or the sample size and demographics [47–49]. Most of these prior studies focused on segmentation, and only one reported agreement with quantitative parameters characterizing the bowel [51]. Several of these studies have noted the substantial time required for manual image annotation, resulting in little available public training data [47, 49–51]. The achievement of similar levels of agreement using visual image annotation may help address these limitations.

### Sources of Error

The gastrointestinal tract is not a perfect cylinder, and some approximation error is expected when modeling it with a constant diameter. Nonetheless, since the caliber of the bowel is common in clinical usage, the diameter was appropriate to annotate in addition to segmentation. Since multiple loops of bowel can be so closely apposed that there is no intervening mesentery, bowel diameter cannot be derived from segmentation. Eye tracking makes annotating diameter feasible ergonomically, since one has a free hand to use to adjust the knob encoding diameter, while adjusting slice location with a mouse in the opposite hand and recording in-plane position based on the gaze location generated by the eye tracker.

Eye trackers may have errors that are heteroskedastic over the space of the monitor or over the time of recording, and must be calibrated for maximal accuracy and precision [52]. Viewing continuous structures such as the gastrointestinal tract on cross-sectional imaging studies may minimize errors by inducing the small eye motions of smooth pursuit rather than the larger eye motions of saccades [53]. The design of the 3D Slicer module was intended to minimize errors by recentering the viewport to use a smaller portion of the monitor and by adjusting calibration with small mouse motions during recording.

### Limitations

This study itself had several limitations. First, the entire length of time required for manual versus visual annotations was not assessed. If the visual annotation is found to be sufficient for downstream applications, an investigation of its efficiency relative to manual annotations will be pursued in future research. Another limitation was that complete blinding was not possible due to project staffing, though manual and visual annotations were separated by at least 1 week. Lastly, manual diameter measurements were only made on thick slice axial reformats, since thin slices were not routinely stored at the investigator’s institution. The performance in other planes will be the topic of future research.

Another limitation was that diagnostic performance for bowel obstruction was not assessed. Neural networks have been applied directly to the detection and characterization of bowel obstruction on abdominal radiographs [54–56] and for the identification of transition points of obstructed bowel on abdominal CT scans [57]. However, the purpose of this study was to generate parametric maps, which may help characterize the severity and etiology of the obstruction, rather than only its presence or absence. The results of this study will enable future investigations of automated detection of bowel obstruction using metrics based on diameter maps [43].

An additional limitation of this study was that there were too few subjects to allow statistical analysis stratified by each relevant clinical or technical feature. The performance of segmentation may depend on these features. Bowel resections, ostomies, or hernias may change the location within a scan at which bowel must be identified. The contents of the peritoneal cavity cause variation in the background from which bowel must be differentiated. Intravenous or enteric contrast may alter the appearance of the bowel itself. These features were balanced between the training and test data sets. Some incorrect predictions of these features are noted in the test data set. However, statistical evaluation will be the topic of future research involving larger data sets.

Another limitation was the low resolution of the normalized data used for training. Training data needed to be resized in both axial and longitudinal dimensions so that a batch could fit within the memory of the single 12 GB GPU available for the study. Patch-based approaches were not investigated, since it was unclear how to measure the diameter of segments that would extend across patches. Fortunately, newer GPUs with more memory will enable the use of higher-resolution training data and of deeper neural networks, from which greater performance is expected. Fortunately, visual annotations were recorded parametrically, so derived bowel segmentations and diameter maps can be reconstructed at higher resolutions. Higher levels of agreement may be possible using manual annotations in addition to visual annotations for training. These efforts will also be the topic of future research.

## Conclusion

In conclusion, eye tracking is a promising technology for visual image annotation for training CNNs in the context of bowel obstruction. Agreement between two repetitions of visual annotation and between visual and manual annotations was good for bowel segmentation and excellent for diameter measurement. Agreement of CNN predictions with manual and visual annotations was moderate for bowel segmentation and diameter measurements, but improved performance may be achieved if limitations are addressed. Whether these levels of repeatability and agreement are acceptable ultimately depends on downstream applications, but the availability of CNN predictions based on visual image annotations will allow the development and validation of such applications.

## Supplementary Information

Below is the link to the electronic supplementary material.

Supplementary file1 (MP4 50108 KB)

## Data Availability

Data that support the findings of this study are available from the author upon reasonable request with restrictions.
